# A single-cell perspective on immunotherapy for pancreatic cancer: from microenvironment analysis to therapeutic strategy innovation

**DOI:** 10.3389/fimmu.2024.1454833

**Published:** 2024-10-30

**Authors:** Rui Wang, Jie Liu, Bo Jiang, Benjian Gao, Honghao Luo, Fengyi Yang, Yuntao Ye, Zhuo Chen, Hong Liu, Cheng Cui, Ke Xu, Bo Li, Xiaoli Yang

**Affiliations:** ^1^ Department of General Surgery (Hepatopancreatobiliary Surgery), The Affiliated Hospital of Southwest Medical University, Luzhou, China; ^2^ Academician (Expert) Workstation of Sichuan Province, Metabolic Hepatobiliary and Pancreatic Diseases Key Laboratory of Luzhou City, The Affiliated Hospital of Southwest Medical University, Luzhou, China; ^3^ General Surgery Day Ward, Department of General Surgery, The Third People’s Hospital of Chengdu, Affiliated Hospital of Southwest Jiaotong University, The Second Affiliated Hospital of Chengdu, Chongqing Medical University, Chengdu, China; ^4^ Department of Radiology, Xichong People’s Hospital, Nanchong, China; ^5^ Department of Oncology, Chongqing General Hospital, Chongqing University, Chongqing, China

**Keywords:** pancreatic cancer, immunotherapy, single-cell technology, tumor microenvironment, predictive biomarkers, personalized treatment

## Abstract

Pancreatic cancer remains one of the most lethal malignancies, with conventional treatment options providing limited efficacy. Recent advancements in immunotherapy have offered new hope, yet the unique tumor microenvironment (TME) of pancreatic cancer poses significant challenges to its successful application. This review explores the transformative impact of single-cell technology on the understanding and treatment of pancreatic cancer. By enabling high-resolution analysis of cellular heterogeneity within the TME, single-cell approaches have elucidated the complex interplay between various immune and tumor cell populations. These insights have led to the identification of predictive biomarkers and the development of innovative, personalized immunotherapeutic strategies. The review discusses the role of single-cell technology in dissecting the intricate immune landscape of pancreatic cancer, highlighting the discovery of T cell exhaustion profiles and macrophage polarization states that influence treatment response. Moreover, it outlines the potential of single-cell data in guiding the selection of immunotherapy drugs and optimizing treatment plans. The review also addresses the challenges and prospects of translating these single-cell-based innovations into clinical practice, emphasizing the need for interdisciplinary research and the integration of artificial intelligence to overcome current limitations. Ultimately, the review underscores the promise of single-cell technology in driving therapeutic strategy innovation and improving patient outcomes in the battle against pancreatic cancer.

## 1 Background

Pancreatic cancer has emerged as a focal point in global medical research due to its exceedingly high fatality rate and the scarcity of treatment options (1–4). This type of cancer’s five-year survival rate is astonishingly low, less than 10%, significantly lower than most other types of cancer (5). These poor survival rates have not changed significantly in nearly 40 years, highlighting the malignancy of pancreatic cancer and the urgency of treatment. One of the primary challenges of pancreatic cancer lies in its almost asymptomatic early stages, leading to a diagnosis at an advanced stage for the majority of patients (6, 7). Furthermore, the difficulty in diagnosing pancreatic cancer and the lack of early diagnosis mean that patients miss the optimal time to receive effective treatment (8, 9). About 70% of pancreatic cancer patients will develop cachexia that cannot be reversed through traditional nutritional support (10). Consequently, researchers aim to improve early diagnosis rates and find more effective treatments to enhance survival and quality of life.

Immunotherapy has shown significant potential in cancer treatment, leveraging the patient’s immune system to recognize and eliminate cancer cells (11). Unlike traditional surgery, chemotherapy, and radiation therapy, immunotherapy activates the immune system to target cancer cells effectively. Although successful in treating cancers like melanoma and non-small cell lung cancer, immunotherapy has lagged in pancreatic cancer due to unique tumor microenvironment and immune evasion mechanisms (12, 13).Pancreatic cancer’s dense desmoplastic stroma hinders immune cell infiltration, a stark contrast to the less dense environments in cancers like breast or prostate cancer (14). Additionally, pancreatic cancers secrete high levels of immunosuppressive cytokines such as TGF-β and IL-10, creating a tumor microenvironment that hinders effective T-cell response (15, 16). The lower mutational burden in pancreatic cancer results in fewer targets for immune recognition compared to other cancers with high mutational burdens (17). Moreover, pancreatic cancer cells express high levels of PD-L1, leading to T-cell anergy and apoptosis (18, 19). However, with in-depth research into the pancreatic cancer tumor microenvironment, scientists have begun to find that adjusting the use of immune checkpoint inhibitors can effectively change the tumor microenvironment, enhancing the immune system’s ability to attack pancreatic cancer cells (20). Furthermore, combining immunotherapy with other treatments, such as targeted therapy and chemotherapy, has also shown potential to improve treatment effects, providing new hope for pancreatic cancer patients and opening new directions for future treatment research (21–23).

Single-cell technology provides a novel perspective for understanding the complexity of pancreatic cancer. This technology enables high-throughput gene expression analysis at the single-cell level, revealing the cellular heterogeneity in the tumor microenvironment (24, 25). Cellular-level heterogeneity is a significant challenge in treating pancreatic cancer, as it leads to varied responses to treatments (26). Single-cell technology has identified distinct subpopulations of cancer-associated fibroblasts that contribute differently to tumor progression and therapy resistance. For instance, Elyada et al. identified fibroblast subtypes with pro-tumorigenic and anti-tumorigenic properties, which has significant implications for targeted therapies. Peng et al. used scRNA-seq(single-cell RNA sequencing) to uncover rare cell populations resistant to conventional therapies, providing new therapeutic targets (27).

Through single-cell technology, researchers can analyze gene expression patterns, functional states, and interactions of different cell types in the tumor microenvironment, crucial for understanding pancreatic cancer’s response to immunotherapy (28). Specifically, this technology helps identify tumor cells evading immune surveillance and immune cells playing key roles in tumor combat, providing a basis for designing new immunotherapy strategies (29). For example, Zhang et al. used single-cell analysis to identify immune cell subsets correlating with better patient outcomes, paving the way for personalized immunotherapy (30). Additionally, single-cell technology can monitor treatment effects by analyzing changes in tumor microenvironment cells before and after treatment, assessing treatment plans’ effectiveness (20, 31–34).

## 2 The revolutionary impact of single-cell technology

### 2.1 Application of single-cell technology in pancreatic cancer research

Single-cell technology allows detailed study of each cancer cell, revealing unknown molecular markers and pathways. For example, through single-cell sequencing technology, researchers have been able to depict the signaling pathway activity map in pancreatic cancer cells, revealing differences between different cell populations (35). These differences are not only present among cancer cells expressing KRAS mutations but also among cells in the tumor microenvironment, such as macrophages and T cells, in terms of their functional states (36). Through these in-depth analyses, researchers have discovered that cells in the tumor microenvironment interact with cancer cells by secreting various factors (such as cytokines and chemokines), which are crucial for tumor growth, metastasis, and response to treatment (37, 38).

### 2.2 Single-cell analysis reveals tumor heterogeneity and immune microenvironment

Single-cell technology reveals not only tumor cell heterogeneity but also the complexity of immune cells in the tumor microenvironment (39). These cells play dual roles in tumor growth and spread. Single-cell sequencing helps identify and classify immune cell subtypes in the tumor and study their interactions with cancer cells through specific molecular pathways. For example, Tregs (regulatory T cells) suppress immune response by expressing molecules like CTLA-4 and TGF-β, facilitating tumor cell escape (40). This insight supports developing therapeutic strategies targeting these immunosuppressive pathways. Clinical trials are exploring inhibitors of CTLA-4 and TGF-β for pancreatic cancer, with early-phase studies indicating that combining these inhibitors with immune checkpoint blockade can enhance anti-tumor immune responses and improve outcomes (41). Similarly, analysis of macrophages in the tumor microenvironment shows that M2-type macrophages promote tumor growth and suppress immune responses by secreting molecules like IL-10 and upregulating PD-L1, aiding tumor cells in evading immune surveillance (42, 43). Therapeutic strategies are being developed to reprogram these M2 macrophages into the pro-inflammatory M1 phenotype, supporting anti-tumor immunity. Preclinical studies show that targeting IL-10 and PD-L1, combined with macrophage reprogramming agents, can reduce tumor burden and enhance treatment efficacy (19). These examples illustrate the crucial role of single-cell technology in revealing the complexity of pancreatic cancer and its microenvironment, providing unprecedented opportunities to understand disease mechanisms and develop new therapeutic methods.

## 3 Single-cell analysis of the pancreatic cancer immune microenvironment

### 3.1 Complexity of the pancreatic cancer immune microenvironment and its impact on treatment response

**Figure 1:**
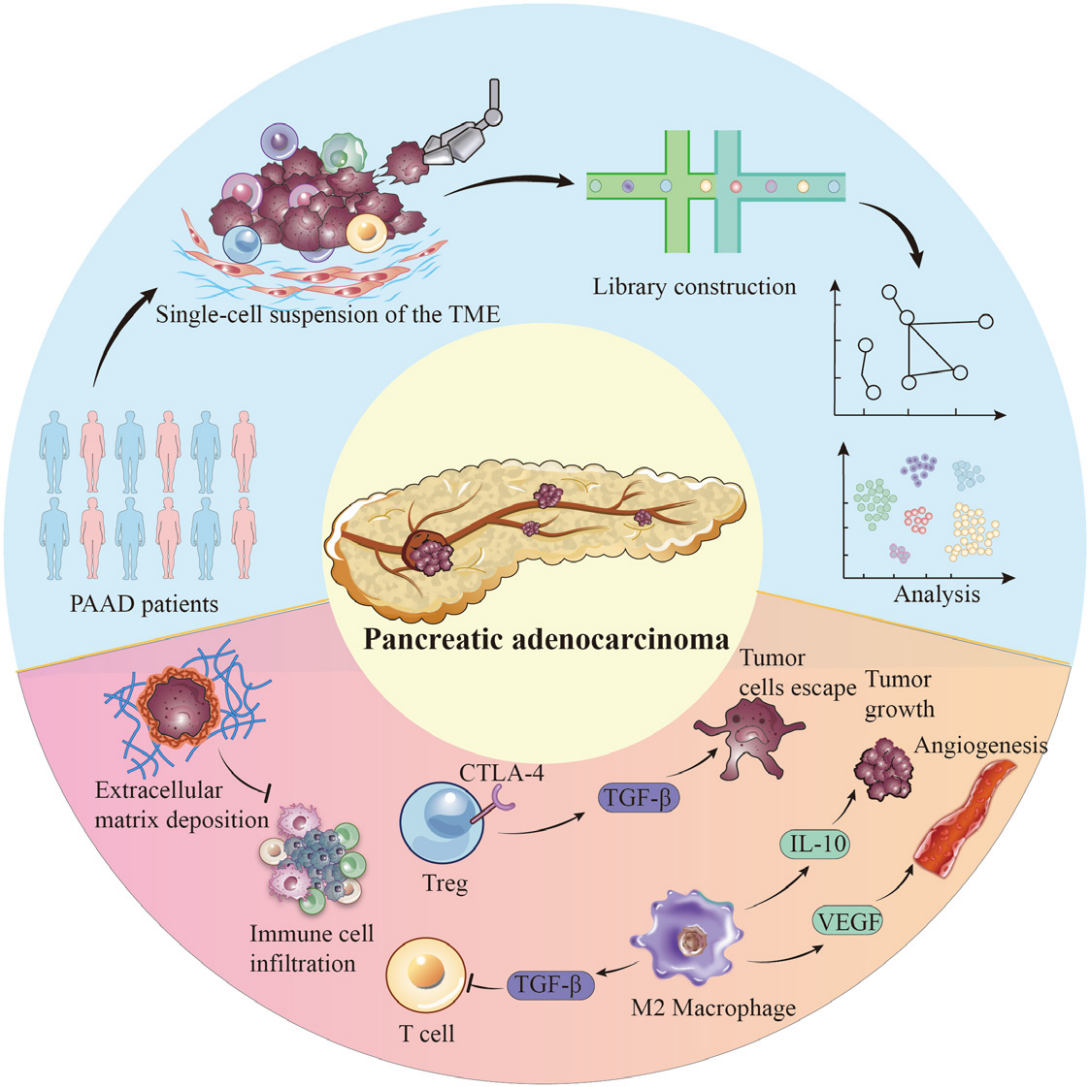
Single-cell perspective of immune cell populations and tumor cell populations in the pancreatic cancer microenvironment.

The pancreatic cancer immune microenvironment is a complex network of tumor cells, immune cells, fibroblasts, and endothelial cells. Their interactions affect tumor development and treatment response. Tregs and TAMs (tumor-associated macrophages), prevalent in the pancreatic cancer immune microenvironment, suppress effector T cell activity and promote immune evasion by releasing factors like TGF-β (44, 45). Particularly, TAMs in pancreatic cancer often exhibit an M2 polarization state, where M2-type TAMs further promote tumor growth and angiogenesis by secreting molecules like IL-10 and VEGF (46–48). Moreover, the fibrotic characteristic of pancreatic cancer, namely the formation of scar tissue and dense deposition of the extracellular matrix, constitutes a physical and biochemical barrier that hinders the infiltration of immune cells. This feature not only reduces the permeability of tumor tissue to drugs but also limits the access and attack capabilities of immune cells to tumor cells (49–51) (**Figure 1**).

### 3.2 How single-cell data can help identify mechanisms of immune therapy response

Single-cell technology, particularly scRNA-seq, provides a powerful tool for deciphering the pancreatic cancer immune microenvironment, enabling researchers to study cellular heterogeneity and complex interactions at an unprecedented resolution (52, 53). Through single-cell analysis of pancreatic cancer patient samples, researchers have identified a group of CD8+ T cells specifically expressing PD-1, which are exceedingly rare in untreated tumors but significantly increase after PD-1/PD-L1 blockade therapy (54, 55). This finding not only confirms the role of PD-1 as an immune checkpoint but also identifies specific cell subpopulations responding to immunotherapy, offering potential for personalized medicine. Further, single-cell sequencing reveals “immune desert” phenomena where some pancreatic tumors have scarce immune cells, actively repelled by specific chemokine and adhesion molecule patterns (56, 57). This rich analysis helps understand the complexity of the pancreatic cancer immune microenvironment and reveals mechanisms of immune therapy response and failure, crucial for designing new therapeutic strategies and achieving personalized treatment (58).

## 4 Predictive biomarkers discovered through single-cell technology

### 4.1 Predictive biomarkers of immune therapy response identified by single-cell analysis

Single-cell technology has revealed key biomarkers predicting immune therapy response in pancreatic cancer. High-resolution scRNA-seq allows precise analysis of cellular heterogeneity in the tumor immune microenvironment, identifying biomarkers like high PD-1 expression on CD8+ T cells, indicating an exhausted state linked to responsiveness to anti-PD-1/PD-L1 therapy (56, 58–60). Clinically, these findings can help personalize therapy, prioritizing patients with high PD-1 levels for anti-PD-1/PD-L1 treatment (61). The M2 polarization state of TAMs, secreting IL-10 and TGF-β, also impacts immune therapy effectiveness (62). Identifying these states through single-cell sequencing can guide combination therapies targeting PD-1/PD-L1 and modulating the macrophage environment to enhance immune response (63). Single-cell data also reveal signaling pathways regulating immune cell activity, providing mechanisms to predict immune therapy outcomes accurately (64–66).

### 4.2 Exploring the potential contributions of these markers to personalized treatment strategies

The predictive markers discovered by single-cell technology provide a substantial scientific basis for personalizing pancreatic cancer treatment. In clinical practice, the presence of these markers can guide physicians in selecting the most suitable immune therapy drugs, optimizing treatment plans, and avoiding the waste of resources on treatments unlikely to be effective. For example, by pre-testing tumor samples for specific phenotypes and functional states of immune cells, doctors can predict the patient’s responsiveness to PD-1/PD-L1 or CTLA-4 inhibitors, thus selectively choosing the treatment method most likely to benefit (67). Additionally, these in-depth insights obtained through single-cell analysis also offer directions for developing new therapeutic targets and strategies (25, 42). For instance, targeting specific subsets of exhausted T cells found in the pancreatic cancer tumor microenvironment, researchers can design novel treatment approaches, such as using biologics to restore these cells’ activity or enhance their tumor-killing capabilities (68). Similarly, therapeutic strategies targeting M2-type TAMs could involve transforming these cells into an anti-tumor M1 polarization state to inhibit tumor growth (29, 69, 70).

## 5 Therapeutic strategy innovation driven by single-cell technology

### 5.1 New immunotherapy strategies developed based on single-cell analysis results

Single-cell technology, particularly scRNA-seq, has driven innovation and precision in pancreatic cancer treatment strategies (25, 53). By analyzing individual cells in the tumor microenvironment, researchers have identified various cell markers and molecular pathways that predict treatment response. These include specific T cell subtypes, immune checkpoint expression patterns, and levels of pro-inflammatory and anti-inflammatory cytokines (54, 71, 72). Based on these findings, new immunotherapy strategies have emerged, including biomarker-guided immune checkpoint inhibitor therapy, targeted cell therapy, and methods to modulate the tumor microenvironment to enhance immune response (73). For example, Chen et al. (2023) found that integrating immune checkpoint inhibitors with therapies targeting the KRAS pathway significantly enhanced immune response in pancreatic cancer models, reducing tumor progression and extending survival compared to single-agent treatments (74). Additionally, single-cell analysis has revealed aberrant signaling pathways that offer new combination therapy opportunities. Combining specific pathway inhibitors with immune therapies, such as PD-1/PD-L1 blockers, can overcome pancreatic cancer’s immune evasion mechanisms, improving treatment outcomes (75, 76). For instance, Lu et al. found that targeting the JAK/STAT signaling pathway, in conjunction with PD-L1 blockade, enhanced CD8+ T cell infiltration and activity in pancreatic tumors, significantly reducing tumor burden (77).

### 5.2 Clinical prospects of single-cell-driven therapeutic strategy innovations

Translating single-cell-driven pancreatic cancer immunotherapy strategies from the laboratory to clinical application presents enormous prospects (42). Personalized treatment plans, based on detailed tumor microenvironment analysis, can improve treatment specificity and effectiveness, reduce unnecessary side effects, and enhance patient prognosis and quality of life (67). However, the clinical implementation of these strategies faces numerous challenges. First, the high costs and technical requirements limit the widespread clinical application of single-cell technology (78). Second, although single-cell analysis can provide detailed information about the tumor microenvironment, translating these complex data into effective treatment plans remains a challenge (79). Furthermore, the safety and effectiveness of these single-cell analysis-based treatment strategies need to be verified through clinical trials, a time and resource-intensive process (42). Lastly, the high heterogeneity and complexity of pancreatic cancer mean that even the most advanced treatment strategies may encounter resistance issues, necessitating continuous exploration and optimization of treatment plans (80).

## 6 Future research directions and challenges

### 6.1 Deepening exploration of future directions in pancreatic cancer research with single-cell technology

As single-cell technology continues to advance, the exploration of new markers will remain a focus of future research, especially those that can guide immunotherapy and precision medicine, such as specific subtypes of immune cells or unique molecular patterns of tumor cells (25). Additionally, future pancreatic cancer research may emphasize analyzing cell heterogeneity and interactions to reveal subtle differences in cancer development and treatment response (42). Specifically, the combined use of single-cell transcriptomics, single-cell epigenetics, and single-cell proteomics is expected to provide new perspectives on complex molecular mechanisms in pancreatic cancer that are not yet fully understood (25). For example, by analyzing the epigenetic states of different cell types in pancreatic cancer tumors, researchers can identify key epigenetic modifications that promote tumor growth and immune evasion, exploring targeted treatment strategies against these modifications (53). Additionally, Single-cell analysis of cell-cell communication, such as exosome-mediated signaling, will reveal immune suppression network mechanisms, offering strategies to disrupt these networks (81). The application of single-cell technology will not be limited to studying pancreatic cancer cells themselves but will also extend to researching non-tumor cells in the tumor microenvironment, such as immune cells, fibroblasts, and endothelial cells (80, 82). For instance, detailed analysis of immune cell diversity and functional states will uncover immune surveillance evasion mechanisms, discovering new immune checkpoints or immunoregulatory molecules as therapeutic targets (52, 54).

### 6.2 Challenges and acceleration of pancreatic cancer treatment progress through interdisciplinary research

Interdisciplinary research, including AI and gene editing technology, plays a crucial role in advancing single-cell studies. AI, especially deep learning, has great potential in processing and analyzing large-scale biological data (83, 84). AI can predict treatment responses at the cellular level, helping design suitable treatment plans for individual disease characteristics. For instance, AI can analyze single-cell data to identify cell subpopulations sensitive to chemotherapy or targeted therapy, customizing compounds or treatment methods (23, 85, 86). Combining single-cell technology and AI can also analyze treatment failure mechanisms, identifying cell subpopulation evolution patterns and underlying molecular mechanisms to develop strategies overcoming treatment resistance (87–89). Gene editing, combined with precise molecular targets from single-cell analysis, offers new treatment strategies by editing specific genes in certain cell subpopulations (90, 91).However, integrating single-cell technology, AI, and gene editing faces challenges, including technical complexity, ethical issues, data processing, and clinical application aspects. Ensuring data security and patient privacy is crucial when handling sensitive medical data (92–94). Technological advancements may exacerbate medical resource disparities, necessitating international cooperation and policy-making to improve access to advanced technologies in low-income countries (95, 96). Ethical considerations include the implications of applying gene editing technologies like CRISPR-Cas9 to human applications, which have sparked widespread ethical debates. A study in Nature (2022) highlighted potential ethical concerns with gene editing, including effects on offspring, off-target effects, and social equity issues (97). Ensuring the safe and ethical use of these advanced technologies remains a significant challenge that must be addressed.

## 7 Discussion

Single-cell technologies have fundamentally transformed our comprehension of the tumor microenvironment (TME) in pancreatic cancer, uncovering significant heterogeneity not just among different tumors but also within the same tumor. This detailed analysis has illuminated the complex interplay between various immune cell types, such as T cells, macrophages, and MDSCs, within the TME, each playing distinct roles in tumor immunity (71). Through identifying specific subpopulations of immune cells that either support or hinder the anti-tumor immune response, such as specific T cell exhaustion profiles and myeloid cell markers, these technologies provide insights that could lead to the development of novel therapeutic strategies aimed at enhancing immunotherapy efficacy (98).

Exploring single-cell mechanisms offers unparalleled opportunities to optimize therapeutic strategies in pancreatic cancer. By elucidating the molecular signals and pathways that control the behavior of individual cell types within the TME, researchers can develop targeted therapies to alter the immune landscape in favor of an effective anti-tumor response. This includes strategies like targeting the PD-1/PD-L1 axis in T cells, which, despite limited efficacy, remains promising (99). Single-cell analyses not only shed light on resistance mechanisms but also guide the integration of immunotherapies with other treatments (67, 100), such as chemotherapy, aiming to surmount existing therapeutic barriers. Furthermore, these analyses hold the potential for personalizing medicine by identifying biomarkers that predict a patient’s response to immunotherapy, thereby tailoring treatments to maximize efficacy and minimize the risk of ineffective treatment exposure (101).

However, it is crucial to acknowledge the current limitations of single-cell technology and their impact on clinical applications. One significant limitation is the technical complexity and high cost associated with single-cell sequencing, which can limit its widespread adoption in clinical settings, particularly in low-resource environments. High-throughput single-cell analyses require sophisticated equipment, substantial computational resources, and specialized expertise, posing accessibility challenges (102). Additionally, the vast and complex data generated from single-cell technologies necessitate advanced computational methods and bioinformatics tools for proper analysis and interpretation, leading to challenges in data integration and standardization essential for translating single-cell insights into clinically actionable information (103). Variability in sample preparation and sequencing protocols can introduce biases, affecting reproducibility and reliability of results (102). Another limitation is the potential for incomplete cellular profiling, where certain cell types or states may be underrepresented or missed entirely, leading to gaps in understanding the TME, especially when identifying rare cell populations crucial for tumor progression and treatment response (104).

Despite these limitations, integrating single-cell technologies with advanced computational methods and machine learning algorithms promises to enhance our understanding of the TME, predict treatment outcomes more accurately, and uncover novel therapeutic targets (25). Interdisciplinary collaboration, uniting biologists, clinicians, and data scientists, is pivotal for translating complex biological insights into actionable, clinically viable treatments. Single-cell technologies thus stand as an indispensable tool in the quest to improve pancreatic cancer patient outcomes through more effective, personalized immunotherapeutic strategies, marking a significant step forward in the fight against this challenging disease (105).
